# Influence of Reactive Chain Extension on the Properties of 3D Printed Poly(Lactic Acid) Constructs

**DOI:** 10.3390/polym13091381

**Published:** 2021-04-23

**Authors:** Maria-Eirini Grigora, Zoi Terzopoulou, Konstantinos Tsongas, Panagiotis Klonos, Nikolaos Kalafatakis, Dimitrios N. Bikiaris, Apostolos Kyritsis, Dimitrios Tzetzis

**Affiliations:** 1 Digital Manufacturing and Materials Characterization Laboratory, School of Science and Technology, International Hellenic University, 14 km Thessaloniki, 57001 N. Moudania, Greece; megrigora@ihu.edu.gr (M.-E.G.); k.tsongas@ihu.edu.gr (K.T.); 2 Laboratory of Polymer Chemistry and Technology, Department of Chemistry, Aristotle University of Thessaloniki, 54124 Thessaloniki, Greece; pklonos@central.ntua.gr (P.K.); dbic@chem.auth.gr (D.N.B.); 3 Department of Physics, Zografou Campus, National Technical University of Athens, 15780 Athens, Greece; akyrits@central.ntua.gr; 4 Institute of Electronic Structure and Laser, Foundation for Research and Technology (FORTH), 70013 Heraklion, Greece; kalafatakisn@iesl.forth.gr; 5 Department of Materials Science & Technology, University of Crete, 70013 Heraklion, Greece

**Keywords:** additive manufacturing, 3D printing, biobased polymers, poly(lactic acid), chain extender

## Abstract

Fused deposition modeling (FDM) is currently the most popular 3D printing method, where thermoplastic polymers are predominantly used. Among them, the biobased poly(lactic acid) (PLA) governs the FDM filament market, with demand higher than supply, since not all grades of PLA are suitable for FDM filament production. In this work, the effect of a food grade chain extender (Joncryl ADR^®^ 4400) on the physicochemical properties and printability of PLA marketed for injection molding was examined. All samples were characterized in terms of their mechanical and thermal properties. The microstructure of the filaments and 3D-printed fractured surfaces following tensile testing were examined with optical and scanning electron microscopy, respectively. Molecular weight and complex viscosity increased, while the melt flow index decreased after the incorporation of Joncryl, which resulted in filaments of improved quality and 3D-printed constructs with enhanced mechanical properties. Dielectric spectroscopy revealed that the bulk properties of PLA with respect to molecular mobility, both local and segmental, were, interestingly, not affected by the modifier. Indirectly, this may suggest that the major effects of the extender are on chain length, without inducing chain branching, at least not to a significant extent.

## 1. Introduction

Additive manufacturing (AM), also known as 3D printing, allows for the fabrication of fully personalized designs with geometrical complexity, while decreasing the use of tools, the cost during prototyping steps, and the fabrication time [1]. No requirement for molds, costly tools, milling, or sanding processes is the main reason why it is a low-cost production method. Even though the basic methods of thermoplastic forming (extrusion, injection molding, thermoforming) are the mainstream in the polymers industry, 3D printing is more efficient, timesaving, and minimizes the use of raw materials [2]. During the past few decades, different applications of 3D printing have been investigated in various industries due to the interest of scientists, engineers, and the medical community [3]. The extensive use of 3D printing in recent years has increased the interest in fused deposition modeling (FDM), which is a widely used method for 3D printing, due to good efficiency, easy material deposition, and low costs [4].

In the last few decades, the development of novel materials for 3D printing has attracted considerable interest from researchers, tailored to special applications [1]. In FDM technology, most of the filaments are not environmentally friendly due to the utilization of petroleum-based materials for fabrication. Consequently, during 3D printing, toxic substances could be released that have negative impacts on health and the environment [5]. Over the last few years, bio-based polymers, such as poly(lactide acid) (PLA), have attracted much attention in 3D printing, replacing petroleum-based polymers due to their availability from renewable and environmentally friendly resources, as well their outstanding properties such as compostability, high tensile strength and modulus [6,7]. PLA is one of the most frequently used bio-based thermoplastic polyesters, which can be either amorphous or semi-crystalline [8,9]. In fact, after the recognition of PLA as an ideal 3D printing filament, demand is larger than supply, which has resulted in a lack of availability. The monomers of PLA can be derived from corn, sugar beets, sugarcane, or wheat, which are biological and renewable sources [10,11,12,13]. The application of neat PLA in FDM technology increases year by year [14]. The mechanical response of 3D-printed PLA was found better than injection-molded PLA [15]. However, PLA has some disadvantages, such as poor thermal stability; brittleness; and, in some cases, low molecular weight, which make it unsuitable for some large-scale end uses. To overcome these limitations and improve its properties, the addition of nanoparticles in PLA or blending it with other bio-based or oil-based polymers are often utilized [6,16,17].

Chain extenders under the trade name Joncryl have been successfully used to improve the compatibility of polymer blends with PLA, such as PLLA/PDLA or poly(butylene adipate-co-terephthalate)/poly(lactide) (PBAT/PLA), resulting in better mechanical and thermal properties [18,19,20,21,22]. Epoxide chain extenders have also been able to improve the layer-to-layer adhesion strength of 3D-printed PLA layers [23] and impart printability in some polymer blends (PLA/polyamide 11) [7,24], PBAT/PLA [25,26], as well as PLA-based composites [27].

The aim of the present work is to improve the printability of medium molecular weight PLA ($\bar{M_{n}}$ = 75,300 g/mol), which is not suitable for FDM, by increasing its molecular weight with reactive chain extension. To the best of our knowledge, there is no literature to account for 3D printing with FDM technology for PLA 3052D and Joncryl ADR 4400 without the addition of other polymers, natural fibers, or nanoparticles. Thus, the aim of the present work is to investigate the role of the multi-functional epoxide Joncryl ADR-4400 as a reactive chain extender in the PLA 3052D matrix for fused deposition modeling (FDM) applications. PLA constructs were manufactured using three different additive contents (1, 2, and 3 wt%) of Joncryl in the polymer matrix. This paper examines the effects of the chain extender, aiming to improve printability and increase the molecular weight of PLA, as well as its mechanical, thermal, and molecular mobility properties.

## 2. Materials and Methods

### 2.1. Materials

The polylactide (PLA-Ingeo^TM^ Biopolymer 3052D, NatureWorks) used in this study was kindly donated by Plastika Kritis S.A. (Iraklion, Greece) in the form of solid flakes, which is designed for injection molding. In the current paper, the term “PLA” is used to represent PLA grade 3052D. The polymeric chain extender Joncryl ADR^®^ 4400 was in the form of solid flakes and was supplied by BASF (Ludwigshafen, Germany). It has an epoxy equivalent weight of 485 g/mol and a weight-average molecular weight of 7100 g/mol. In this paper, the term “Joncryl” is used to represent Joncryl ADR^®^ 4400.

### 2.2. Fabrication of PLA Filaments and 3D Printed Specimens

PLA was dried under vacuum at 40 °C overnight before mixing and extrusion. Joncryl flakes were first crushed into powder with a Thomas milling machine (Thomas Scientific, Swedesboro, NJ, USA) for 5 min. Then, the dried PLA and the predetermined amount of Joncryl powder were manually mixed by stirring and then placed in the single screw extruder. Three different concentrations of Joncryl were tested: 1 wt%, 2 wt% and 3 wt%. In this paper, the terms PLA/J1, PLA/J2, PLA/J3 are used to represent the blend of PLA with 1 wt%, 2 wt%, and 3 wt% Joncryl, respectively. PLA/Joncryl filaments were prepared using Filament Maker-Composer 350 (3devo, Utrecht, The Netherlands). Each blend of PLA with Joncryl was extruded into a 1.75 mm diameter filament with temperatures ranging from 170 °C to 190 °C. The deviation of the filament thickness was 5 μm. PLA/Joncryl samples for dynamic mechanical analyses (DMA), broadband dielectric spectroscopy (BDS), and mechanical testing were printed utilizing a XYZ da Vinci SUPER 3D printer (New Kinpo Group, New Taipei City, Taiwan). The specimens for DMA, BDS, and compression testing were designed through XYZmaker 3DKit and converted into stereolithography (STL) file format. Cylindrical specimens of 12.5 mm diameter and 25 mm height were fabricated for compression testing. The dimensions of the 3D-printed specimens for DMA analysis were 40 × 6 × 2 mm and their geometry was rectangular. The dimensions of the tensile test specimen were 40 mm (gage length) × 5 mm (width) × 3.8 mm (thickness). All the samples were fabricated with 100% infill. Τhe tensile specimens were designed using SolidWorks2020 and converted into stereolithography file format. Slicing was performed in XYZprint and G-code was generated for the 3D process.

A stainless steel nozzle with 0.4 mm diameter was used in the XYZ da Vinci SUPER. The extruder deposits material layer by layer from this nozzle onto a building plate. The printing head was set to 30 mm/s, the printing bed temperature to 45 °C, and the nozzle temperature in the range from 205 to 215 °C. A concentric pattern was used for the outer two layers (shell), while a rectilinear infill pattern was introduced by the XYZ software with a ±45 degrees angle, which is considered typical for the most FDM 3D printers. A layer height of 0.2 mm was applied. The infill percentage was chosen as 100%. A cylindrical plate with 25 mm diameter and 3.2 mm height was printed in order to preliminarily examine the 3D printing performance of each filament at four different extruder temperatures. The best 3D printing quality was noticed at 205 °C, 210 °C, 210 °C, and 215 °C for the filaments of PLA, PLA/J1, PLA/J2, and PLA/J3, respectively. Tensile specimens were oriented flatwise in the XY plane. Images of the 3D-printed specimens are shown in Figure S1, in the Supplementary Materials section.

### 2.3. Physicochemical Characterization

#### 2.3.1. Gel Permeation Chromatography

The molecular weight of the materials was determined using gel permeation chromatography (GPC) with a Waters 600 high-performance liquid chromatographic pump, Waters Ultrastyragel (Milford, MA, USA) columns HR-1, HR-2, HR-4E, HR-4, and HR-5, and a Shimadzu RID-10A (Kyoto, Japan) refractive index detector. For the calibration, 9 polystyrene (PS) standards of molecular weight between 1 and 300 kg/mol were employed. The prepared solutions had a concentration of 12 mg /mL, the injection volume was 150 μL, and the total elution time was 50 min.

#### 2.3.2. Melt Flow Index (MFI)

The MFΙ of the filament melts were measured at 205 °C, 210 °C, 210 °C, and 215 °C for the filament of PLA, PLA/J1, PLA/J2, and PLA/J3, respectively, using a melt flow quick index meter (CEAST, Turin, Italy) according to the ASTM standard D 1238-04 and ISO standard 1133 (load 2.16 kg).

#### 2.3.3. Microscopy

A stereoscope was used to observe the filaments. Photographs were captured using a Jenoptik (Jena, Germany) ProgRes GRYPHAX Altair camera attached to a ZEISS (Oberkochen, Germany) SteREO Discovery V20 microscope and Gryphax image capturing software.

The fracture surfaces of the 3D-printed tensile test specimens were characterized with scanning electron microscopy (Phenom ProX, ThermoFisher Scientific, Waltham, MA, USA). The images were acquired from the cross-section area of the tensile tested 3D-printed specimens. Samples were mounted onto double adhesive conductive carbon tabs (TED Pella, Redding, CA, USA) on an aluminum stub (placed in a charge reduction holder) without coating and scanned at an accelerating voltage of 10 kV.

#### 2.3.4. Linear Viscoelastic Measurements (LVE)

The dynamic storage (*G*′) and loss (*G*″) moduli for the four samples were measured at temperatures above their melting point by means of a strain-controlled rheometer (ARES 2kFRT-N1 from TA, New Castle, DE, USA) equipped with a force rebalance transducer. When the temperatures needed to access the terminal flow regime (where $G^{\prime \prime }\propto \;\omega^{1}$ and $G^{\prime }\propto \;\omega^{2}$) were too high, creep compliance measurements at lower temperatures were conducted and the data were transformed into dynamic moduli [28]. For the latter measurements, a stress-controlled rheometer (MCR 702 from Anton Paar, Graz, Austria) was utilized. The samples were heat pressed (at 170 °C for about 20 min) into discotic specimens using a home-made mold under dynamic vacuum. For the measurements, stainless steel parallel plates with a diameter of 25 mm were used, and the sample thickness (gap) was about 1.5 mm. Samples were loaded onto the bottom plate of the rheometer connected to the motor and melted to remove thermal and mechanical history. Then, the upper plate connected to the transducer was loaded until the sample completely filled the gap. A dynamic strain sweep test was performed prior to any measurement in order to determine the linear viscoelastic regime. Subsequently, dynamic frequency sweep tests were performed in the range of 0.1 to 100 rad s^−1^ and for strain amplitudes within the linear viscoelastic regime. The thermal expansion of the tools was measured independently and taken into account during the measurements. The measurement protocol ensured that the possibility of thermal degradation of the samples [29,30,31,32] was minimized. The time temperature superposition principle was successfully applied for all the four samples. The Arrhenius equation $a_{T}=exp\left[\frac{-E_{a}}{R}\left(\frac{1}{T}-\frac{1}{T_{ref}}\right)\right]\;$, where *E*_a_ is the activation energy for flow and *R* is the global constant of ideal gases, was used to fit the horizontal shift factors (α_T_) used to obtain the master curves. Some tiny vertical shifting was performed to account for the density variation of the samples. The limited temperature window due to crystallization temperature and possible chemical degradation did not allow for measurements over an extended temperature range, hence, only three temperatures are measured. Additionally, the temperature difference from the glass transition temperature being about 100 °C led us to use the Arrhenius equation to fit the horizontal shift factors. Eventually all the master curves were shifted to a reference temperature so that *T*_ref_ = *T*_m_ + 20 °C, thus allowing for direct comparison of the rheological quantities for all the samples.

#### 2.3.5. Fourier Transform Infrared Spectroscopy (FTIR)

The chemical structures of neat PLA and PLA/J were determined with FTIR spectroscopy. FTIR spectra of the samples were recorded utilizing an FTIR-2000 (Perkin Elmer, Waltham, MA, USA). A small amount of filament was hot-pressed for a few seconds in order to create thin films for testing. The spectra were collected in the range from 400 to 4000 cm^−1^ at a resolution of 4 cm^−1^ (total of 16 co-added scans), while the baseline was corrected and converted into absorbance mode.

#### 2.3.6. Thermal Analysis

DSC measurements were performed employing a Perkin Elmer Pyris 6 DSC apparatus Waltham, MA, USA) calibrated with indium and zinc standards in order to examine the crystalline state of the samples. About 5 mg of each sample were placed in sealed aluminum pans and heated up from 30 to 200 °C with a heating rate of 20 °C/min, (N_2_, flow rate 50 mL/min).

Thermogravimetric analysis (TGA) of all prepared filaments was carried out using a SETARAM SETSYS TG-DTA 16/18 instrument (Setaram, Lyon, France). The mass loss and its first derivative curves of the prepared materials were obtained. Samples (2 ± 0.2 mg) were placed in alumina crucibles, while an empty alumina crucible was used as a reference. Then, the samples were heated from room temperature to 600 °C in an 8.3 × 10^−7^ m^3^ sec^−1^ flow of N_2_ at a heating rate of 20 °C min^−1^.

Dielectric spectroscopy (DS) was employed on the 3D-printed specimens for polymer dynamics. Measurements were carried out by means of a Novocontrol DS setup (Novocontrol (GmbH, Montabaur, Germany). The samples were inserted between finely polished brass electrodes (20 mm in diameter) along with thin silica spacers (50 μm in thickness) and melted therein on a hot plate, forming a sandwich-like capacitor. The complex dielectric permittivity, ε* = ε′−i·ε″, was recorded isothermally as a function of frequency in the range from 10^−1^ to 10^6^ Hz and in the temperature range between −120 and 100 °C, heating at steps of 5 and 10 K, depending on the process followed.

### 2.4. Mechanical Characterization

#### 2.4.1. Tensile Testing

The tensile properties of the PLA/J blends were investigated by testing 3D-printed specimens of each different content of Joncryl in PLA. The tensile properties of the samples were measured at room temperature (23 °C) by utilizing a M500-50AT (Testometric, Rochdale, UK) universal testing machine that was equipped with a 50 kN load of cell. The 3D printing specimens were tested at a constant rate of 5 mm / min until the specimen was ruptured. At least three specimens were tested for each sample. The dimensions of the tensile test specimens were 40 mm (gage length) × 5 mm (width) × 3.8 mm (thickness).

#### 2.4.2. Compression Testing

For the compression test, cylindrical specimens of 12.5 mm diameter and 25 mm height were prepared through the FDM 3D printing technique and the test was accomplished using an M500-50AT (Testometric, Rochdale, UK) universal testing machine with a 50 kN load of cell. At least three specimens were tested for each sample. The compression properties of the samples were measured at room temperature (23 °C).

#### 2.4.3. Nanoidentation Testing

The mechanical performance of neat PLA and PLA/Joncryl filaments was investigated through nanoindentation testing. The hardness of the samples was measured with a dynamic ultra-microhardness tester DUH-211 (Shimadzu Co., Kyoto, Japan) using a 100 nm radius triangular pyramid indenter tip (Berkovich-type indenter). During the indentation test, a controlled load (P) with a peak load of 30 mN was applied through a diamond tip on the surface of the filaments. This peak load was held for 3 s. The indentation depth was recorded as a function of load. Subsequently, the indenter was unloaded to a load of zero. The maximum indentation load was applied to the indenter during the creep time. The modulus and hardness were obtained as the average value of ten measurements.

#### 2.4.4. Dynamic Mechanical Analysis (DMA)

Dynamic mechanical analysis was performed using a Diamond DMA dynamic mechanical analyzer (Perkin Elmer, USA). The dimensions of the 3D-printed specimens were 40 × 6 × 2 mm, and their geometry was rectangular. Three samples were manufactured for each blend. They were measured in a 3-point bending mode with 1 Hz oscillation frequency. The study was conducted at a temperature range from 30 °C to 90 °C, the heating rate was 3 °C min^–1^. The storage modulus (*E*′), loss modulus (*E*″), and loss factor (tan δ) were recorded as a function of temperature.

## 3. Results and Discussion

### 3.1. Effect of Chain Extender of Molecular Weight, Filament Fabrication and Printing

The mechanical properties and crystallization behavior of PLA heavily depends on its molecular weight [33]. The use of Joncryl in reactive extrusion significantly increases the molecular weight and the melt strength of the polymer. The reaction involves covalent bond formation between the hydroxyl group of PLA and the epoxide group, which results from the ring opening of the epoxide. It is known that the addition of a chain extender increases the molecular weight of the polymers [21], but high concentrations can lead to branched structures [18]. Therefore, in this work, small concentrations of Joncryl were used. Table 1 presents the molecular weight, PDI, and MFI of neat PLA and PLA/J. After extruding the PLA solid flakes into PLA filament, a small decrease in the molecular weight due to thermal degradation was observed. The addition of Joncryl increased the molecular weight of PLA by ~50, 70, and 80% for 1, 2, and 3 wt% chain extender, respectively, thus protecting it from degradation [8,34,35]. The MFI of pure PLA filament was 4.29 g/10 min, which was higher than the PLA/J blends. This indicates that the addition of the Joncryl causes a decrease in the fluidity of the PLA matrix. The MFI value of PLA/J1 considerably decreased to 1.48 g/10 min, which is a 65.6% decrease compared with neat PLA. A substantial decrease in the fluidity of the PLA/J2 up to 91.5%, approximately, as compared with pure PLA could also be noticed. This result indicates that the material became more rigid, as well as more viscous, by the addition of Joncryl, raising the quality of fused filament fabrication of polylactic acid-based composites.

The morphology and average diameter of the filaments was examined with the help of stereoscopic images, and the values as a mean of 10 measurements are included in Table 1. The optimum diameter was 1.75 mm, to comply with the specifications of the 3D printer. While the filaments of PLA had a smaller diameter, with an average value of 1.36 mm, all blends with Joncryl had an average value of about 1.7 mm, which is closer to the ideal diameter of 1.75 mm, as well as smaller standard deviations. The filaments were more uniform in morphology with the addition of Joncryl as a chain extender in the PLA [36].

**Figure 1:**
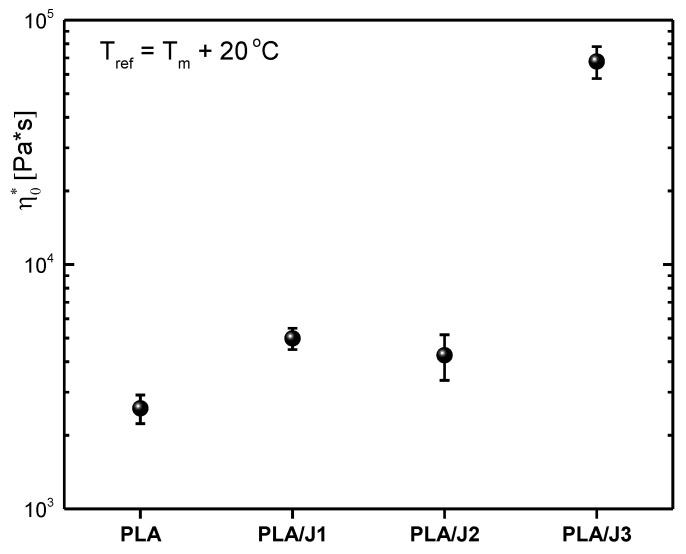
Zero shear (complex) viscosity for all the samples calculated at the same temperature distance from the *T*_m_, as derived from the fitting of the data with the Carreau model.

Figure 1 depicts the values of the zero-shear complex viscosity η*=G*ω for the four samples studied when data were shifted to the same temperature distance from the calorimetric *T*_m_. One can see the clear increase of the complex viscosity for the PLA/J1 and PLA/J2 samples compared to the neat PLA melt. These two samples display nearly the same complex viscosity within experimental error. For PLA/J3, the complex viscosity increased significantly, which could be a result of branching caused by the relatively large amount of Joncryl in the polymer [18]. The Carreau model [37] was employed to fit the data and reveal the value of the zero-shear (complex) viscosity. The dynamic moduli, and especially the storage modulus G^’(data not shown here), indicate that the quadratic dependence on frequency expected for Newtonian flow was not reached directly after their crossover. Data display a frequency dependence to about the 1.8 power against the reduced frequency and eventually reach the quadratic frequency dependence at the lowest frequency regime, typical behavior for polydisperse samples. In the case of the PLA/J1 and PLA/J2 samples, the former frequency regime was accessed in terms of creep compliance measurements and the transformation of the data into dynamic moduli [28]. Creep compliance measurements facilitated extending the spectra to lower frequencies that were not accessible in terms of frequency sweeps, since the temperatures needed would lead to thermal degradation of the samples.

### 3.2. Thermal Properties

**Figure 2:**
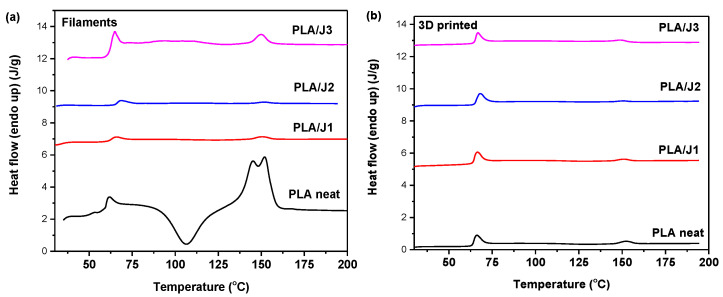
DSC traces of the (**a**) PLA filaments and the (**b**) PLA 3D-printed specimens during heating at 20 °C/min.

The DSC traces of the PLA filaments and 3D-printed constructs are shown in Figure 2, and the thermal characteristics extracted from the traces are included in Table 2. All samples received were amorphous, showing glass transition at 61–62 °C and cold crystallization and subsequent melting. The cold crystallization and melting enthalpies were almost equal, revealing the amorphous nature of the as-received filaments. The endothermic transition that appeared near the end of the glass transition was caused by molecular relaxation [38]. *T*_cc_ increased with the incorporation of Joncryl because polymer crystallization was hindered as the molecular weight increased [39], the *T*_m_ slightly decreased, and the double peaks disappeared, suggesting the formation of only one type of crystals (α- ordered phase). After 3D printing, cold crystallization was hindered, likely due to the porosity of the specimens, which is believed to prevent the growth of crystals [27], which is more evident for the PLA neat and PLA/J1 samples. In general, the thermal transitions of the samples before and after 3D printing were not significantly affected.

**Figure 3:**
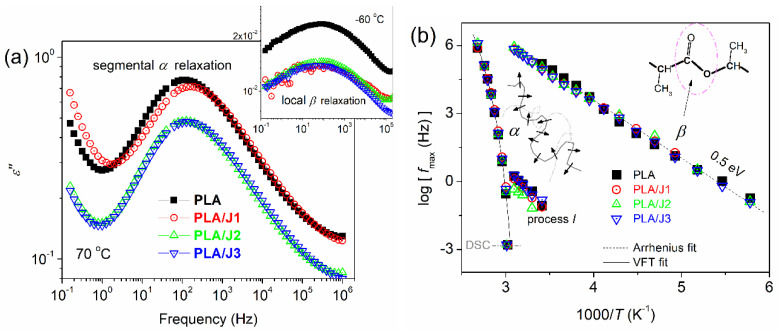
(**a**) Comparative DS spectra for the imaginary part of dielectric permittivity, ε″, (dielectric loss) against frequency at 70 °C and (inset) −60 °C, representative of the segmental and local dynamics. (**b**) Molecular dynamics (time scale) map for all samples. The inset schemes of (**b**) describe the origins of the recorded processes. The additional process I was necessary for the fitting-analysis.

To further check the almost unchanged calorimetric *T*_g_, and, moreover, to study the impact of Joncryl on local and segmental mobility, DS measurements were performed [40]. Representative data are shown in Figure 3 in the form of dielectric losses, *ε*″. Results are shown at *T* = 70 °C (>*T*_g_), demonstrating the dielectric analogue of glass transition, namely the so-called *α* relaxation and, next to that, results at *T* << *T*_g_, for the more localized mobility of PLA (*β* relaxation, inset schemes in Figure 3) [41,42].

Both types of mobility, local and segmental, seemed to exhibit similar time scales independently of composition. The data were further analyzed in terms of known models (e.g., Havriliak–Negami) [40,43] and, therefore, the overall dynamics map was constructed (Figure 3b). Local and segmental processes were almost unchanged by the modification (*M*_n_), in time scale, strength, and degree of cooperativity. This suggests that the modifier resulted in a unique increase of chain length (*M*_n_) rather than branching, at least not to a large extent. The DS recordings on the *α* process supplement those of the calorimetric *T*_g_. Both the calorimetric and dielectric *T*_g_ were almost identical upon the chain extending, as could be expected, since, as in all cases, the *M*_n_ was far above the threshold for entanglements. The results are interesting also from the point of view of basic physics, as the bulk-like properties of PLA were unaffected by Joncryl.

The thermal stability of the pure PLA and PLA/J filaments was studied using TGA. TGA curves were recorded to examine the effect of the chain extender on the thermal stability of PLA. The mass loss and DTG curves of PLA neat and PLA/J in the form of filaments are shown in Figure S2 and the thermal degradation characteristics in Table 3. The thermal degradation of the pure PLA and PLA/J blends occurred in a single step. The degradation of PLA started at 373.8 °C, which is in full accordance with what was reported in the literature [8,44]. PLA/J started to degrade at slightly lower temperatures, but, according to the DTG graph of Figure S2b, the peak appeared at practically the same temperature. According to the manufacturer, Joncryl 4400 is thermally stable up to 320 °C [45], therefore, the additional mass loss that occured in that temperature range (Figure S2a inset) and caused the reduction of the *T*_o_ is due to the degradation of the Joncryl.

### 3.3. Effect of Chain Extender on the Chemical Structure of PLA

**Figure 4:**
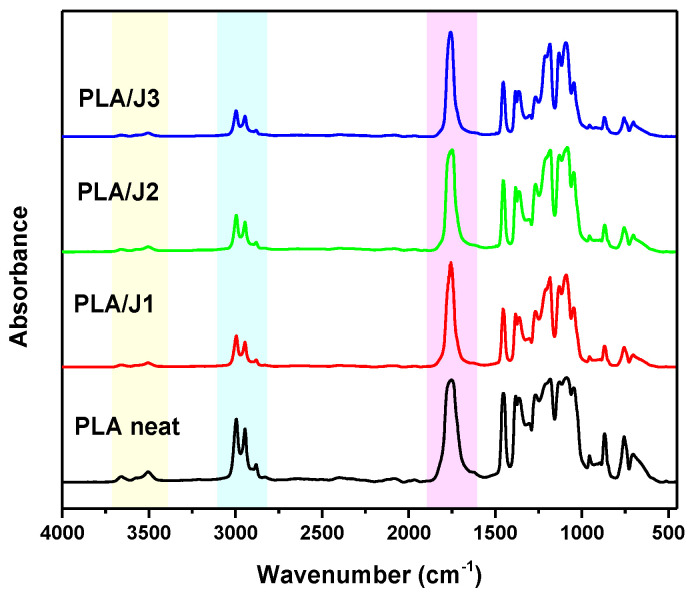
FTIR spectra of neat PLA and PLA/J.

The chemical structure of the filaments and the interactions between the chain extender and PLA were examined by FTIR spectroscopy. Figure 4 illustrates the FTIR spectra of the PLA and PLA/J blends in the range of 4000–500 cm^−1^. In the FTIR spectrum of PLA, the peaks at 3700–3400 cm^−1^ correspond to O–H bending vibrations, while the peaks in the range of 3040–2860 cm^−1^ correspond to C–H stretching. The peaks at 1752 cm^−1^ and 1456 cm^−1^ are assigned as the C=O stretching and –CH_3_ asymmetric bending, respectively. Overlapped peaks between 1270 and 1086 cm^−1^ could be attributed to the stretching vibration of the –C–O–C– groups of PLA. Moreover, the peak at 866 cm^−1^ is due to O–CH–CH_3_ and the peak at 757 cm^−1^ to the wagging vibrations of CH_3_.

Joncryl 4400 is a food grade, multifunctional epoxy chain extender, recommended for PLA, among other polyesters. The reaction that occurs during reactive blending consists of the ring-opening of the epoxides towards carboxyl or secondary hydroxyl groups, which create new covalent bonds and increase the molecular weight [39,46,47,48].

After the incorporation of Joncryl, the characteristic FTIR bands of PLA appeared in the same wavenumbers, confirming that it did not interrupt the structure of PLA’s backbone [49]. The peaks of the hydroxyl and carboxyl groups significantly decreased in intensity due to their reaction with Joncryl and the subsequent chain extension. The ratio of the intensity of the bands of the carbonyls (1752 cm^−1^) to the methyl group bands (3040–2860 cm^−1^) also increased, witnessing the formation of new carbonyl bonds between the carboxyl end groups of PLA and the hydroxyl groups of Joncryl.

### 3.4. Mechanical and Thermomechanical Properties

#### 3.4.1. Mechanical Characterization through Nanoindentation, Tensile and Compression Testing

**Figure 5:**
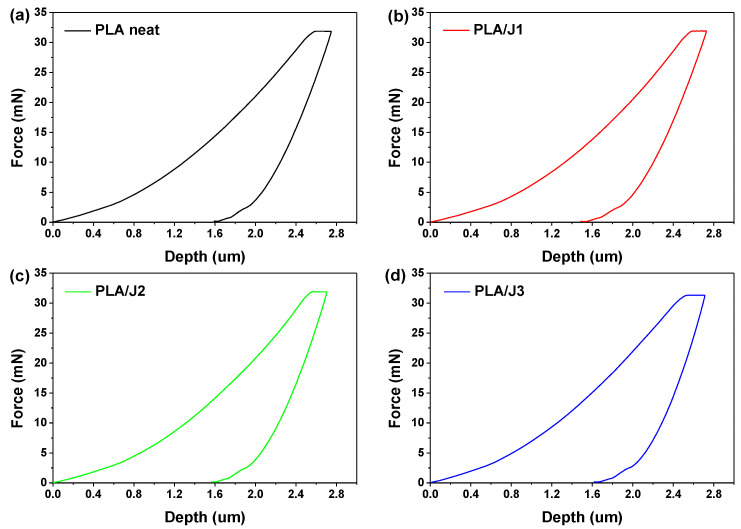
Typical load–depth nanoindentation curves for (**a**) PLA neat, (**b**) PLA/J1, (**c**) PLA/J2, and (**d**) PLA/J3.

The mechanical behavior of the neat PLA and PLA/J specimens was determined using nanoindentation, tensile, and compression testing. In Figure 5, the load–depth curves are illustrated for neat PLA and PLA/J specimens as measured from the nanoindentation tests. The maximum indentation depths were quite similar for all the PLA specimens and were approximately between 2.60 and 2.88 μm. The range of nanoindentation depth was 2.73 to 2.88 μm for neat PLA, 2.72 to 2.81 μm for PLA/J1 samples, 2.60 to 2.65 μm for PLA/J2 samples, and 2.83 to 2.87 μm for PLA/J3 samples.

**Figure 6:**
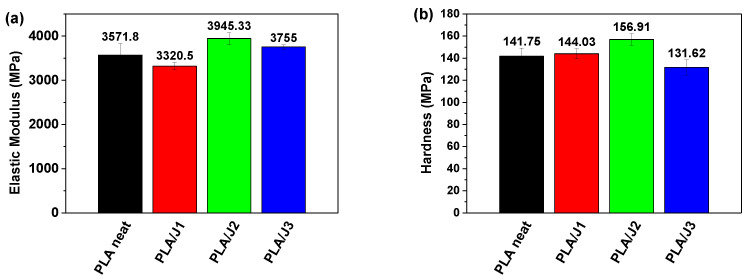
Comparison of (**a**) elastic modulus and (**b**) hardness.of neat PLA and PLA/J filaments.

The values of hardness and elastic modulus of neat PLA and PLA/J filaments were determined based on previous work [50,51,52,53,54,55]. Figure 6a,b show the average values of hardness and elastic modulus depending on Joncryl concentration obtained from nanoindentation testing of PLA/Joncryl filaments. The elastic modulus for pure PLA filament was 3571 MPa and the hardness was 141.7 MPa. The addition of 2 wt% Joncryl increased the modulus to 3721 MPa, which is an approximately 4.2% increase, while the addition of 3 wt% Joncryl increased the modulus to 3972 MPa which is an approximately 11.2% increase as compared to neat PLA. Despite that, a 7% decrease was noticed with the addition of 1 wt% Joncryl. Furthermore, the hardness also increased at around 1.6% and 6.3% by the addition of 1 wt% and 2 w.t%, respectively, while a 7.1% decrease occurred for 3 w.t% Joncryl. The hardness values for 1 wt%, 2 w.t%, and 3 w.t% Joncryl were measured to be 144.02 MPa, 150.57 MPa, and 131.62 MPa, respectively.

**Figure 7:**
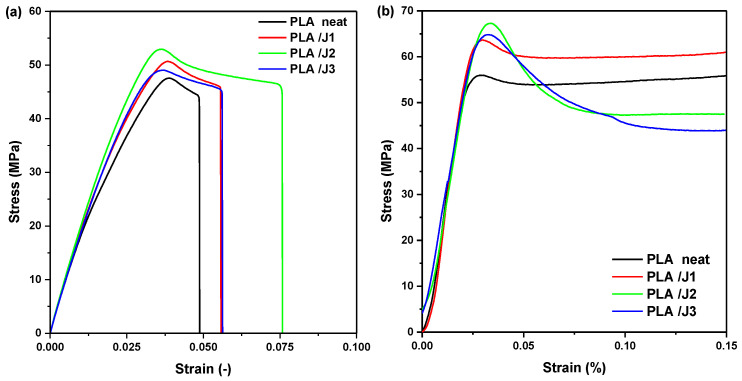
Representative stress–strain curves for (**a**) tensile and (**b**) compression specimens of neat PLA and PLA/J.

Figure 7a presents the stress–strain curves of PLA and PLA/J specimens obtained from tensile tests. In all curves there is a linear elastic zone from which the elastic modulus was determined. Beyond this zone there is a maximum point, which is easy to identify. The maximum stress was used to determine the tensile strength of the PLA/J 3D-printed samples. The specimens were loaded until breaking. It could be noticed that the presence of Joncryl improved the tensile properties of PLA. The best performance was achieved by using 2 wt% of Joncryl in PLA blend. The results of the tensile testing of PLA and PLA/J 3D-printed specimens showed that the elongation can be modified by the addition of Joncryl. The neat PLA had a maximum stress of 47.57 MPa, approximately. The addition of 1 wt% Joncryl into PLA increased the maximum stress to 50.67 MPa, which is an approximately 6.5% increase as compared to neat PLA. The significant increase in maximum tensile stress of 11.3% was obtained with the addition of 2 wt% Joncryl, which corresponds to 52.95 MPa, compared to pure PLA. Moreover, the addition of 3 wt% Joncryl increased the maximum stress to 49.05 MPa which is an approximately 3.1% increase. A general improvement in the maximum tensile strength of the PLA/Joncryl blends could be seen. This could be due to the increased molecular weights [8,56]. A decrease of 8.2% could be noticed for PLA/J3 as compared with PLA/J2. The reason for this might be a high-stress concentration from Joncryl in PLA [7]. The results of the tensile properties are higher in the case of the 2 wt% Joncryl in PLA blend. In terms of elongation, Joncryl 2 wt% slightly increased the ultimate strain from 4.9 to 7.6% (slightly higher ductility) compared to neat PLA, while a larger area was enclosed under the stress–strain curve, indicating a small increase in toughness.

Figure 7b shows the stress–strain curves of PLA and PLA/Joncryl 3D printing samples obtained from compression tests. It can be noticed that the addition of Joncryl can significantly improve the compression properties of 3D-printed specimens compared with neat PLA. The neat PLA had a maximum stress of 56.36 MPa. The addition of 1 w.t% Joncryl in PLA increased the maximum stress to 63.64 MPa, which is around a 12.9% increase as compared to neat PLA. Moreover, the addition of 2 w.t% Joncryl increased the maximum compression stress to 67.26 MPa, which is an increase of 19.34%. The addition of 3 w.t% Joncryl increased the maximum stress to 64.83 MPa, which is an approximately 19.34% increase. A 4.31% decrease could be noticed in PLA/J3 as compared with PLA/J2.

Table 4 presents the summary of the results of the maximum stresses of the tensile and compression testing, and the comparison of the elastic moduli of nanoindentation, tension, and compression tests of neat PLA and PLA/J specimens. The mechanical testing of four specimens demonstrated that the addition of the multi-functional epoxide Joncryl ADR-4400 improved the mechanical behavior of PLA. Although a decrease in the mechanical properties of PLA/J3 could be noticed, which might be due to a high concentration of chain extender in PLA.

The tensile Young’s modulus of neat PLA was up to 2005 N/mm^2^. Comparing PLA/J with pure PLA, the Young’s modulus of PLA/J1, PLA/J2, and PLA/J3 were 2.24%, 9.73%, and 4.74% higher than PLA, respectively, due to the increase in the reactive agent content. In this research, the tensile strength of pure PLA (3052D) was up to 47.57 MPa. The value of tensile strength of neat PLA using in this research was 12.47% lower than the PLA (4032D), which is suitable for FDM. The addition of 2 wt% Joncryl increased the tensile strength up to 52.95 MPa. The maximum tensile strength of PLA/J1, PLA/J2, and PLA/J3 was visibly 6.52%, 11.31%, and 3.11%, respectively, compared with the neat PLA. The maximum compression strength of PLA/J1, PLA/J2, and PLA/J3 was visibly 12.92%, 19.34%, and 15.03%, respectively, compared with the neat PLA. The reason for this is that Joncryl increased the molecular weight of PLA, which is expected after using epoxides as chain extenders for alipharomatic polyesters [57]. It can be noticed that the values of the Young’s modulus in tensile and compression testing, as well as the maximum tensile and compression stress, followed the same trend. In comparison, the Young’s modulus of PLA/J1, PLA/J2, and PLA/J3 was 12.92%, 19.34%, and 15.03%, respectively, compared with the neat PLA. However, there was an obvious difference in the values of the elastic moduli by nanoindentation and tensile testing. This is reasonable because the nanoindentation tests were implemented on a filament form of PLA and PLA/J, while tensile testing was performed on 3D-printed specimens. It is known that 3D printing processes affect the crystallinity of the material through the deposition process. In FDM, the filament melts for several seconds in the nozzle of the 3D printer before being extruded out. During extrusion, the polymer chains were oriented inside the nozzle due to the pressure drop [48,49]. Moreover, the lack of adhesion between the deposited layers and the presence of trapped air in 3D-printed objects might be the reason why the values of tensile and compression Young’s modulus were lower compared with the results of nanoindentation testing.

**Figure 8:**
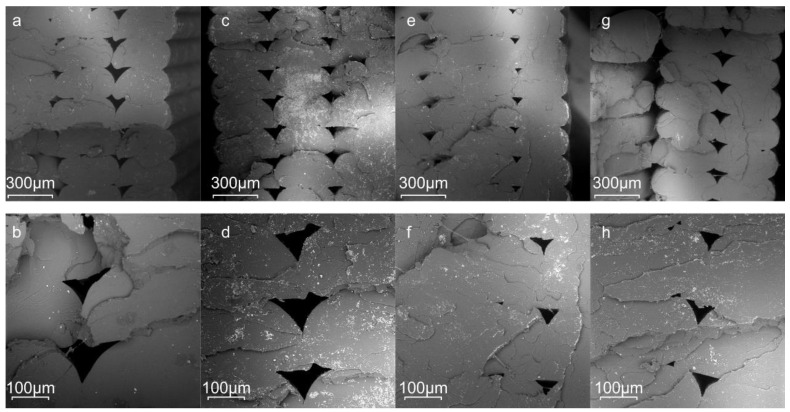
SEM microstructure of the cross section (outer shells) of tensile fracture surface at low and high magnifications for (**a**,**e**) PLA neat, (**b**,**f**) PLA/J1, (**c**,**g**) PLA/J2, and (**d**,**h**) PLA/J3 materials.

**Figure 9:**
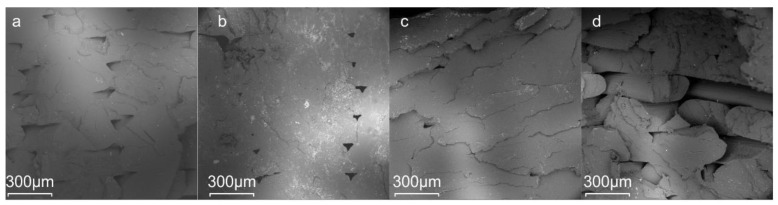
SEM microstructure of the cross section (infill area) of the tensile fracture surface for (**a**) PLA neat, (**b**) PLA/J1, (**c**) PLA/J2, and (**d**) PLA/J3.

Figure 8 shows SEM micrographs at different magnifications of the fracture surfaces from the tensile samples manufactured by the FDM technique. These images show a representative tensile tested microstructure surface of the pure PLA sample and PLA/J blends. Almost symmetric voids are generally created in between the deposited print shells, which seem to be larger in size in the case of neat PLA and PLA/J1. For some specimens, these voids coalesce to create cracks as characteristically shown from the vertically oriented crack in Figure 8d. The highest strength and stiffness obtained from the tensile tests in the case of PLA/J2 could be partly justified from the smaller voids along with the chain extension effect of the 2 wt% Joncryl. Figure 9 shows the infill of the fractured tensile specimens. The void formation is barely noticed in the case of PLA/J2 within the severely deformed 3D-printed struts, while the neat PLA and PLA/J1 showed a similar but worse response in terms of void distortion and fracture behavior. On the contrary, the cross-section of the PLA/J3 showed significant crack propagation between the struts. Clearly, the presence of voids leads to high-stress concentration areas at these particular zones during tensile testing, leading to random crack formation.

#### 3.4.2. Dynamic Mechanical Analysis (DMA)

**Figure 10:**
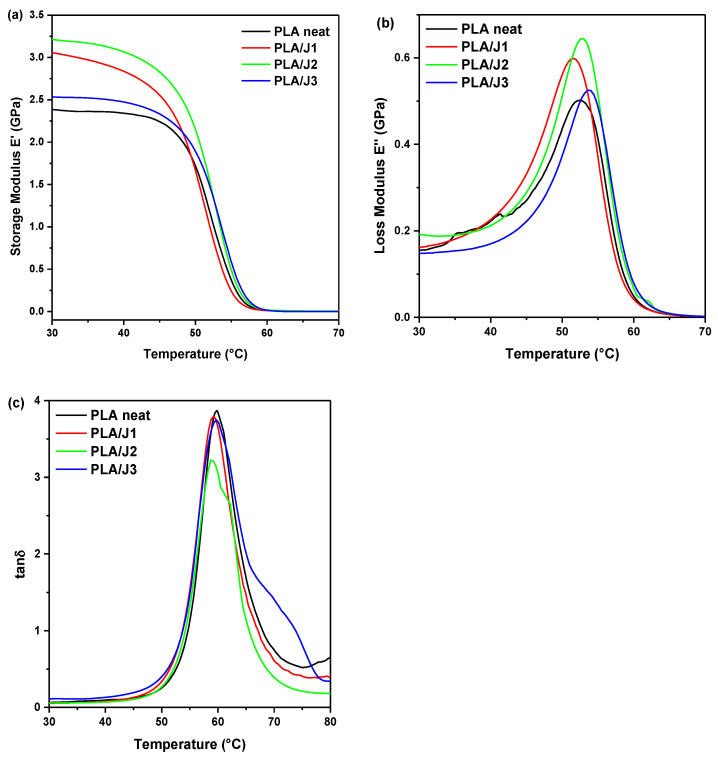
DMA results of the 3D-printed specimens of neat PLA and PLA/J: (**a**) storage modulus *E*′, (**b**) loss modulus *E*″, and (**c**) loss factor tanδ as a function of temperature.

The values of storage, loss moduli, and tangent δ have been measured through DMA of the 3D-printed specimens of PLA neat and PLA/J. The storage modulus $E^{\prime }$ defines the energy stored in the PLA and the PLA with the chain extender specimens due to the applied strain, which corresponds to the stiffness of the specimens. The loss modulus $E^{\prime \prime }$ is proportional to the storage modulus $\left(E^{\prime }\right)$ and (tanδ) values, and is related to the energy dissipation mechanisms involved in the materials under study. The results of the DMA of the 3D-printed specimens of PLA neat and PLA/J are given in Figure 10. As shown in Figure 10a,b, the addition of Joncryl in PLA results in minor changes in the storage (*E*′) and loss (*E*″). There was a minor increase in storage modulus at 30 °C when the Joncryl concentration is increased, especially in the case of PLA/J1 and PLA/J2. The storage modulus of PLA/J1 and PLA/J2 showed an improvement of 29.34% and 35.46%, respectively, compared to neat PLA. At higher temperatures, and especially after 45 °C, the response was similar for all specimens under study. Notably, the highest stiffness was attained with the addition of 2 wt% Joncryl in PLA as compared to 1 wt% Joncryl. This can be attributed to the increase of the molecular weight as discussed previously and as reported in another study as well [58]. It is also believed that the addition of Joncryl in PLA, and especially 2 wt%, has an effect on the adhesion strength between the 3D-printed layers, as seen in the previous sections from the marginal increase of the mechanical tests, as well as from the obtained DMA results. Accordingly, the loss modulus of PLA/J1 and PLA/J2 showed an improvement of 19.37% and 28.48%, respectively, compared to neat PLA, as shown from the peaks at temperatures of 50–55 °C. The tangent δ represents the ratio between the dissipated energy and the elastically stored energy, which is given by the relationship $E^{\prime \prime }/E^{\prime }$. It measures the energy used to bend the PLA and PLA/J specimens during DMA testing and is dissipated directly into heat [59]. The glass transition temperature (*T*_g_) can be deduced from the peak of the tanδ curve and is a function of temperature as seen in Figure 10c. The neat PLA was found to have *T*_g_ values of 59.9 °C, while for PLA/J1, PLA/J2, and PLA/J3 those values were 59.2 °C, 58.7 °C, and 59.6 °C, respectively. In general, insignificantly small changes in the tanδ values were observed among the different specimens.

## 4. Conclusions

In this paper, PLA (3052D) and PLA/J filaments with different content of Joncryl were successfully manufactured and 3D-printed through the fused deposition modeling process to desirable shape and size specimens. Three different contents of Joncryl (1 wt%, 2 wt%, 3 wt%) in PLA 3D-printed specimens were thoroughly characterized with physicochemical and mechanical testing methods. The addition of Joncryl into a PLA matrix increased the molecular weight, melt flow index, and complex viscosity, while thermal stability remained unaffected and cold crystallization was hindered. Therefore, the filaments were fabricated with improved quality, while providing 3D-printed constructs with enhanced mechanical properties compared to neat PLA. Molecular mobility, both local and segmental, was not affected by the Joncryl modifier, as measured by dielectric spectroscopy. This may indicate that the main outcome of the extender influenced the chain length, without inducing chain branching, at least not to a significant extent. Overall, the mechanical test results revealed that the best mechanical performance in terms of elastic modulus and hardness of the PLA material was achieved in the case of 2 wt% concentration of Joncryl.

## Figures and Tables

**Table 1:** Molecular weight, PDI (GPC), and MFI of neat PLA and PLA/J and the average filament diameter.

Sample	$\bar{M_{n}}\;(g/\mathrm{mol})$	$\bar{M_{w}}\;(g/\mathrm{mol})$	*M*_p_ (g/mol)	PDI	MFI (g/10 min)	Average Filament Diameter (mm)
PLA flakes	81,700	126,300	102,300	1.5	3.87 ± 0.03	-
PLA filament	75,300	124,400	107,600	1.6	4.29 ± 0.04	1.36 ± 0.03
PLA/J1 filament	92,600	185,000	124,200	1.9	1.48 ± 0.07	1.71 ± 0.02
PLA/J2 filament	124,300	210,900	172,100	1.6	0.37 ± 0.04	1.71 ± 0.01
PLA/J3 filament	127,200	223,300	187,000	1.7	0.37 ± 0.02	1.70 ± 0.01

*M*_n_: number average molecular weight, *M*_w_: weight average molecular weight, *M*_p_: peak maximum molecular mass, PDI: polydispersity index.

**Table 2:** DSC results of the PLA filaments and 3D-printed constructs.

Form	Sample	*T*_g_ (°C)	*T*_cc_ (°C)	*T*_m_ (°C)	*ΔH*_cc_ (J/g)	*ΔH*_m_ (J/g)
Filaments	PLA neat	61.6	106.3	151.9	−27.35	31.28
	PLA/J1	61.3	123.7	150.4	−22.15	24.64
	PLA/J2	62.8	130.7	151.7	−0.7	2.1
	PLA/J3	61.8	126.0	141.9	−1.42	2.09
3D-printed Constructs	PLA neat	63.4	129.4	152.3	−2.7	3.5
	PLA/J1	63.4	126.7	150.7	−2.6	2.0
	PLA/J2	64.8	127.3	150.6	−1.2	0.8
	PLA/J3	64.1	126.0	149.0	−1.0	2.2

**Table 3:** Thermal degradation characteristics of the PLA and PLA/J filaments.

Sample	*T*_o_ (°C)	*T*_d,10%_ (°C)	*T*_p_ (°C)	Residue (%) at 600 °C
PLA neat	373.8	365.9	394.7	1.38
PLA/J1	372.0	366.0	395.3	1.51
PLA/J2	368.9	363.7	394.2	0.20
PLA/J3	368.1	359.6	393.8	1.67

*T*_o_: onset of degradation, *T*_d__,10%_: temperature that corresponds to 10% mass loss, *T*_p_: peak temperature of DTG where degradation occurs at the fastest rate.

**Table 4:** Comparison of the ultimate stresses of tensile and compression testing and the comparison of the elastic moduli of nanoindentation, tension, and compression testing of neat PLA and PLA/J specimens.

Sample	Ultimate Tensile Stress (MPa)	Ultimate Compression Stress (MPa)	*E*_i_ Nanoindentation (N/mm^2^)	*E*_i_ Tension (N/mm^2^)	*E*_i_ Compression (N/mm^2^)
PLA neat	47.57 ± 1.56	56.36 ± 3.07	3571.80 ± 259.87	2005 ± 6.61	3100 ± 10.48
PLA/J1	50.67 ± 0.43	63.64 ± 0.91	3320.50 ± 82.50	2050 ± 9.95	3300 ± 81.69
PLA/J2	52.95 ± 1.05	67.26 ± 0.39	3945.33 ± 134.74	2200 ± 16.02	3450 ± 9.79
PLA/J3	49.05 ± 2.06	64.83 ± 5.47	3755.00 ± 48.05	2100 ± 3.17	3200 ± 18.28

## Supplementary Materials

The following are available online at https://www.mdpi.com/article/10.3390/polym13091381/s1, Figure S1: Images of the PLA/J2 3D-printed specimens. Figure S2: TGA thermograms of the filaments.
