# Supplementary material for: Influence of Reactive Chain Extension on the Properties of 3D Printed Poly(Lactic Acid) Constructs
Source: Polymers (Basel). 2021 Apr 23;13(9):1381. doi: 10.3390/polym13091381 (PMC8123025; doi:10.3390/polym13091381)
Supplement: Supplementary file 1 [file polymers-13-01381-s001.zip › polymers-1173250-supplementary.pdf]

polymers

Supplementary materials

Influence of Reactive Chain Extension on the Properties of 3D Printed Poly(lactic acid) Constructs

Maria-Eirini Grigora [1], Zoi Terzopoulou [2,*], Konstantinos Tsongas [1], Panagiotis Klonos [2,3], Nikolaos Kalafatakis [4,5], Dimitrios N. Bikiaris [2], Apostolos Kyritsis [3] and Dimitrios Tzetzis [1,*]

1 Digital Manufacturing and Materials Characterization Laboratory, School of Science and Technology, International Hellenic University, 14km Thessaloniki, 57001 N. Moudania, Greece; megrigora@ihu.edu.gr (M.E.G.), k.tsongas@ihu.edu.gr (K.T.)
2 Laboratory of Polymer Chemistry and Technology, Department of Chemistry, Aristotle University of Thessaloniki, 54124 Thessaloniki, Greece; pklonos@central.ntua.gr (P.K.); dbic@chem.auth.gr (D.B.)
3 Department of Physics, National Technical University of Athens, Zografou Campus, 15780, Athens, Greece; akyrits@central.ntua.gr
4 Institute of Electronic Structure and Laser, Foundation for Research and Technology (FORTH), 70013 Heraklion, Crete, Greece; kalafatakisn@iesl.forth.gr (N.K.)
5 Department of Materials Science & Technology, University of Crete, 70013 Heraklion, Crete, Greece
* Correspondence: terzozoi@chem.auth.gr (Z.T.); d.tzetzis@ihu.edu.gr (D.T.)

Citation: Grigora, M.-E.; Terzopoulou, Z.; Tsongas, K.; Klonos, P.; Kalafatakis, N.; Bikiaris, N. D.; Kyritsis, A.; Tzetzis, D. Influence of Reactive Chain Extension on the Properties of 3D Printed Poly(lactic acid) Constructs. *Polymers* **2021**, *13*, 1381. https://doi.org/10.3390/polym13091381

Academic Editors: Gianluca Cicala anf Andreia F. Sousa

Received: 22 March 2021
Accepted: 20 April 2021
Published: 23 April 2021

Publisher's Note: MDPI stays neutral with regard to jurisdictional claims in published maps and institutional affiliations.

Copyright: © 2021 by the author. Licensee MDPI, Basel, Switzerland. This article is an open access article distributed under the terms and conditions of the Creative Commons Attribution (CC BY) license (http://creativecommons.org/licenses/by/4.0/).

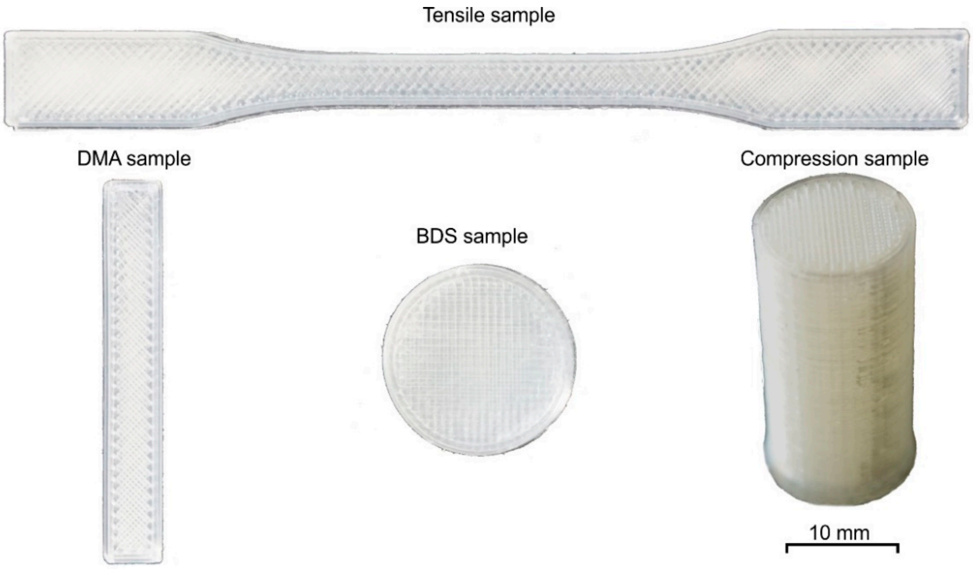

Figure S1. Images of the PLA/J2 3D printed specimens.

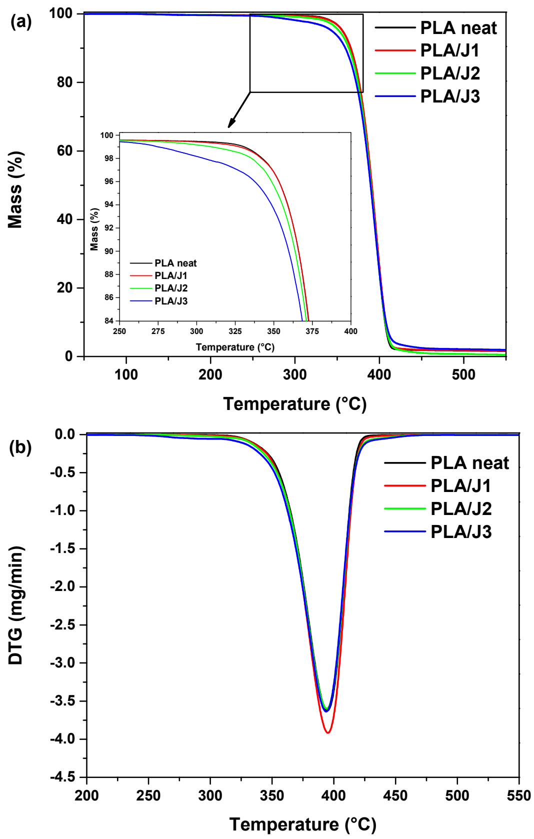

Figure S2. TGA thermograms of the filaments.
